# The regenerative compatibility: A synergy between healthy ecosystems, environmental attitudes, and restorative experiences

**DOI:** 10.1371/journal.pone.0227311

**Published:** 2020-01-07

**Authors:** Matteo Giusti, Karl Samuelsson

**Affiliations:** 1 Department of Building Engineering, Energy Systems and Sustainability Science, University of Gävle, Gävle, Sweden; 2 Department of Geospatial and Computer Sciences, University of Gävle, Gävle, Sweden; Institute for Advanced Sustainability Studies, GERMANY

## Abstract

Urban nature is and will be the most common provider of nature interactions for humankind. The restorative benefits of nature exposure are renown and creating human habitats that simultaneously support people’s wellbeing and ecological sustainability is an urgent priority. In this study, we investigate how the relationship between environmental attitudes and healthy ecosystems influences restorative experiences combining a place-based online survey with geographical data on ecosystem health in Stockholm (Sweden). Using spatial regression, we predict the 544 restorative experiences (from 325 respondents), with people’s environmental attitudes, natural land covers, ecosystem health, and the statistical interactions among these variables as predictors. Our results show that restorative experiences can happen anywhere in the urban landscape, but when they occur in natural environments, the combined levels of biodiversity and ecological connectivity are better predicting factor than the mere presence of nature. That is, healthy ecosystems seem to be more important than just any nature for restorative experiences. Moreover, the statistical interaction between one’s environmental attitudes and natural environments predict almost all restorative experiences better than when these variables are independent predictors. This suggests that there is synergistic compatibility between environmental attitudes and healthy ecosystems that triggers restorative processes. We call this synergy *regenerative compatibility*. Regenerative compatibility is an unexploited potential that emerges when people’s attitudes and ecosystems are aligned in sustainability. We consider regenerative compatibility a valuable leverage point to transform towards ecologically sustainable and healthy urban systems. To this end, we encourage multifaceted policy interventions that regenerate human-nature relationships holistically rather than implement atomistic solutions.

## Introduction

Urban landscapes are and will be the most common human habitat [1]. As a consequence, the benefits to human’s health and wellbeing that nature experiences provide will predominantly occur via human-designed green infrastructure [2]. The restorative effects of nature experiences for human’s wellbeing are well-known [3,4]. Thus, nature in cities has intrinsic ecological and restorative value [5]. However, if cities are to be designed to support both people and the biosphere, it is critical to expose barriers and synergies that exist between healthy urban ecosystems and healthy people. Unveiling such interactions could help to understand, and eventually restore, a relationship between human and ecological functioning that is of great value to promote sustainable futures. The sustainability arena has long discussed the need for a paradigmatic shift to re-align social values and personal preferences with the ecological functioning of the biosphere [6–8]. This shift is required to approach sustainability as a process that supports all forms of life under ever-changing conditions, rather than a static goal that translates into an exercise of efficiency and impact minimisation [9]. Analysing the potential synergy between human and ecological health means exploring an underlying social-ecological system dynamic that might manifest as a self-reinforcing process of sustainable human-nature co-evolution. System dynamics with self-promoting properties are called *regenerative* and they are of central interest for strategies that aim to holistically address the ever-changing target of sustainability [9–11].

Despite substantial scholarly progress, the interplay between urban ecology and human wellbeing is still an open research frontier [12,13]. This is especially true when the benefits provided by urban ecosystems are cultural, subjective, or intangible [14]. However, positive attitudes towards the environment could be key to understand which relations could simultaneously promote healthy ecosystems and healthy people [15]. Evidence shows that accumulated nature experiences have the possibility to shape environmental attitudes [16,17]. However, the human brain also has a proactive role in constructing the experience happening in the moment [18]. Restorative processes are engaged not only when one’s mind is freed from the daily routine, but also when the environment fits one’s purposes and inclinations [4]. The compatibility between one’s attitude towards nature and the kind of ecosystem experienced might therefore be central to restorative and regenerative processes that promote sustainable co-evolution between social and human systems. Thus, the scope of this paper is to explore the compatibility between nature attitudes, ecosystem health and restorative experiences.

In the sections below, we introduce the aim and background of the paper, present our conceptual approach, and then describe the methods used. Lastly, we present the results and discuss how environmental attitudes and healthy ecosystems create useful synergy for public health.

## Aim and background

The aim of this paper is to investigate how the relationship between environmental attitudes and healthy ecosystems influences restorative experiences. We hypothesise that restorative experiences in nature exist when there is compatibility between people’s attitude for nature and the kind of natural environments experienced.

Experiencing nature is proven to provide a variety of positive effects on human bodies and minds (for reviews see [19–21]). These benefits are not limited to interactions with pristine or wild environments. Evidence on restorative experiences has shown that urban natural areas have greater restorative effects on people than in built settings [4,22–24]. This is true even when nature interactions happen at home and workspaces [25], just as 40-second views of green roofs [26], or through technological mediums [27], sounds [28,29] or printed photographs [30]. The urban green infrastructure has undoubtedly shown the potential to restore human health and wellbeing [2] and it is now considered a risk-decreasing solution for psychological and physiological disorders [31,32]

Most of this research relies on the sole presence of natural features to explain the restorative effects of an environment. However, there is growing evidence that suggests that restorative benefits might be depending on the *relationships* between people and environments. For instance, familiarity with the spatial environment and social context are found to be restorative factors in both children [33] and adults [34]. Von Lindern reports that constraints to restorative experiences are both setting-dependent [35] and dependent on one’s professional occupation [36]. Grahn and Stigsdotter [37] show that emotional and social perceptions of urban green spaces relate to restoration from stress. Moreover, Scopelliti and Giuliani [38] suggest that social and affective factors are important features of restorative experiences. Research in environmental psychology further promotes this reasoning. Haga et al. [28] suggest that “it is not the stimulus features *per se* that underpins restoration but instead the meaning that is attributed to the stimulus.” In this latter study, white-noise is restorative for mental fatigue only when participants are either told or believe that they are listening to a waterfall, rather than to the sounds of an industrial environment.

The evidence above suggests that restorative processes associated with nature exposure might not be explained solely by environmental features, but they might also involve the positive expectations and associations that one holds with the environment. If we are to create cities that support both the biosphere and human health, it is thus crucial that these expectations and associations align with ecological functioning. Healthy ecosystems provide the ecological services that underpin human existence [12] and their degradation is an impending threat for humanity [39,40]. Healthy ecosystems are connected, biodiverse, and resilient [41,42]. A healthy ecosystem is a sustainable ecosystem [43]. This is the desired endpoint of any environmental management and the ambition of any sustainable civilization. At the same time, positive attitudes towards nature and social values constitute the psychological foundation to promote environmental conservation and sustainable practices [44–47]. Unveiling the relationship between these two drivers of sustainable living can aid strategic interventions to promote healthy populations living in healthy ecosystems.

## Conceptual framework

The conceptual approach behind this paper is relational (i.e. transactional) rather than interactional [48]. That means that the focus of this research is *not* to analyse how psychological attributes or environmental features separately contribute to restorative experiences, but it is to understand the restorative value emerging from their relationships. This conceptual approach expects the restorative value of nature experiences to emerge from the simultaneous interplay of psychological attributes, people’s actions, and physical environments. Hence, we explore the patterns of the restorative phenomena in relational terms rather than through cause-effect mechanisms.

This approach is at the basis of the theory of affordances and embodied ecosystems. Affordances are defined as ‘relations between abilities to perceive and act and features of the environment’ [49]. By combining the theory of affordances and embodied cognition, Raymond et al. [50] describe ecosystem services as emerging from multilevel relationships between elements of mind, body, culture, and environment. This is different from the concept of ecosystem services in itself. The directionality of the concept of ecosystem services is problematic because it disentangles the role and implications that people have in many ecosystems [51]. Differently, embodied ecosystems suggest that the benefits that nature provides to humans emerge from the ever-changing patterns of relationships between humans’ mind, body, culture, and environment [50]. In this paper, we hypothesise that the restorative value emerging from nature experiences could be similarly understood by relationships between one’s mind and the environment.

In restorative literature relational approaches seem to be increasingly prominent. As von Lindern [35] notes “the idea that restorative processes depend only on environmental characteristics is too simplistic.” According to attention restoration theory, a restorative environment is one that not only allows escaping one’s routine (i.e. being away), being fascinated by many things (i.e. fascination), being immersed and engaged with it (i.e. extent), but also one that is compatible with people’s purpose [4,52]. The latter characteristic is the one of most interest to this study. As Kaplan [4] notes: “there should be compatibility between the environment and one’s purposes and inclinations. In other words, the setting must fit what one is trying to do and what one would like to do. Compatibility is a two-way street.” Compatibility is truly a relational property of restorative environments. Similarly, the relational theory of affordances suggests that an area is suitable for a person if it affords exactly what the person wants to find and do [53]. What a person brings in the interaction with the environment, whether an ability to act or an expectation, might be a core contributing factor for the restorative process to occur and be actualised. The language in some papers about restorative environments reflects this relational position by avoiding the term “restorative environments” in favour of “environments typically relied on for restoration” [35]. However, in the literature on restorative experiences, compatibility has been often overlooked in favour of studying recovering from mental fatigue, stress, or other psychological conditions [52]. A relational approach to restorative processes is therefore not necessarily novel, but so far under-developed and yet crucial to align healthy ecosystems, environmental attitudes, and restorative benefits. In this study, we adopt a relational approach to cover this ground and explore how the compatibility between healthy ecosystems and environmental attitudes associates with restorative processes.

## Methods

### Participants and procedure

All participants of the study (N = 325) are voluntary respondents of an online Public Participatory GIS survey called “Var är ditt Stockholm?”, which in English translates to “Where is your Stockholm?”. The survey is designed to capture people’s positive or negative experiences that consistently occur to them in the Stockholm county. The focus of the survey is not on nature or restorative experiences alone, but on positive and negative experiences in the city of Stockholm more broadly.

To promote awareness about the survey among the inhabitants of Stockholm, the authors participated in an architectural art exhibition (“Experiment Stockholm”), spread the information through a Facebook page and a Twitter account, and contacted several municipalities within the Stockholm county to advertise the study on local newspapers and notice boards. The survey is published online after several pilot-runs among researchers living in Stockholm. The survey is provided in both Swedish and English and takes about 8 minutes to complete. Data is collected for about eight months: from September 2015 to May 2016.

Participants begin answering the survey by marking on a digital map the location where they have reoccurring positive or negative experiences. Afterwards, they qualify their experiences by selecting one or more qualities among a list of 19 attributes (for full details see [54]). Only after the attributes of the experience have been recorded, respondents are asked to respond to eight items about their attitude towards nature (see section below) and provide basic demographic information (i.e. age group and gender).

The online data collection does not ensure a representative sample of the Stockholm population, but it ensures a large dataset of geocoded information that can be used to explore city life from many different angles. The relations between urban features and all positive or negative experiences reported is the subject of a previous study [55]. Since in this study we are specifically interested in restorative nature experiences, only positive experiences are analysed. Within this subgroup, seven attributes are indicators of restorative experiences. Thus, only experiences with these indicators are analysed in this study (see section below).

### Indicators of restorative experiences

In the survey, seven indicators are used to qualify restorative experiences: *escaping one’s routine*, *being relaxed*, *being mindful*, *feeling safe*, *feeling immersed in the place*, *being fascinated*, *being oneself*. These indicators represent different aspects of restorative experiences. Escaping one’s routines, feeling immersed in the place, being fascinated, and being oneself are indicators of the classic attributes of restorative experiences: being away, fascination, coherence, and compatibility [4]. These indicators are also part of the ‘perceived restorativeness scale’ [56]. Feeling safe is an attribute used in this survey because it is considered in the literature to be a potential constraint for restorative experiences [33,57]. Lastly, being relaxed and being mindful are included because they indicate recognised feelings of restoration [58] and because they are indications of stress recovery [3]. In this study, experiences are considered restorative when at least one of these indicators is present.

### Indicators of environmental attitudes

The survey uses eight statements that measures different aspects of one’s attitude towards nature (see Appendix A for details on each statement). These statements are indicators of: *enjoyment of nature*, *empathy for animals*, *domination over nature* (reversed item), *identification with nature* (two indicators), *environmental awareness*, *sense of responsibility for nature*, and *environmental concern*. Environmental attitude is not an easy construct to evaluate, but existing literature and validated psychometric scales have achieved great levels of reliability [59]. Enjoyment of nature is a recognised indicator of Nature Relatedness Scale [60] and empathy for animals and environmental awareness are essential components of Connection to Nature Index [61]. The desire to dominate over nature is a reverse attribute of the widely used New Environmental Paradigm [62] and identification with nature is a central component of the Environmental Identity scale [63]. Lastly, sense of responsibility is a measure of Love and Care for Nature [64] and environmental concern is used in the Environmental Concern scale [65]. Respectively, each of these indicators is shown to contribute to pro-environmental intentions or actions, and they can collectively be considered a representation of positive environmental attitudes. Thus, we term this collection of indicators *environmental attitudes (EA)*. Respondents are asked to answer each statement of EA using a Likert scale from 1 (disagree completely) to 10 (agree completely). Participants’ EA is calculated as the average of these answers.

### Natural land cover, presence of nature, and ecosystem health

In this study, we use public geographic data to create three maps of nature in Stockholm: *natural land covers (NLC)*, *nature presence (NP)*, and *ecosystem health (EH)*.

First, the NLC map is produced by using the Swedish Environmental Protection Agency’s land cover data [66] provided at a 10 m resolution. This GIS map is created by aggregating 16 natural land covers into six complementary categories: open wetland, arable land, open vegetated land, deciduous forest, coniferous forest and mixed forest. Remaining non-natural land covers are merged into a single category: non-natural. This data manipulation results in a map with seven dichotomous variables: six for natural land covers and one for non-natural.

Second, the NP map has a dichotomous classification in which all natural land covers of the NLC map are merged, to distinguish any kind of natural land cover in the landscape from non-natural land covers.

Lastly, the EH map is an ecological network map produced by Stockholm municipality in 2015, provided at a 2 m resolution. For this map, multi-criteria analysis is used to combine the ecological networks of coniferous forest, broadleaf forest, and wetlands according to biotope quality, patch size, and degree of connectivity in the ecological network (for details see [67]). Biodiversity estimations for wetlands based on on-the-ground reports are also included as a criteria. The resulting variable is a score from 0 to 5 that reflect ecological connectivity and biodiversity. EH values are added to attributes of experiences by calculating the average score within a 50 meter buffer from each experience.

### Data analysis

All experiences from the survey that contain details about the respondents’ age, gender, and EA, are analysed. On this dataset we first perform some descriptive analysis. We explore through histograms the demographic composition of our sample with respect to age groups and genders, and how EA differs between the genders. We also explore what proportions of restorative experiences occur in natural environments and at which levels of EH. Secondly, we analyse the hypothesis that restorative experiences in nature are a function of the health of an ecosystem and one’s attitude towards nature. Through stepwise model selection of logistic regressions, we identify the model that best describe each restorative attribute. That means that the choice of variables to predict each kind of restorative experience is carried out systematically by comparing a sequence of statistical regression models. Akaike Information Criterion (AIC) scores are used to compare model fit and at each step of the process the model with lowest AIC is recognised as better fitting for the data. The end result of this process is the identification of the most-fitting model for each kind of restorative experiences. Gender and age groups are included in the models as control variables, and robustness of results is assessed by the range of odds ratios when adjusting or not adjusting for these terms.

In this study, we choose to investigate how the relation between EA and EH predict restorative experiences rather than exploring how restorative experiences can be formative to EA or contribute to EH. This is because the structure of our dataset does not fit the purpose of evaluating only restorative experiences in nature since the online survey do not force restorative experiences to occur only in natural environments. Additionally, although the survey asked about reoccurring experiences, our data is not longitudinal. Each data point represent only one experience and so cannot be assumed to be sufficiently powerful to represent one’s EA.

We include spatial error terms in the models that correct for unmeasured spatial effects [68]. A spatial error term reflects the spatial autocorrelation among residuals, i.e. how similar the residual of a measurement is compared to those geographically close to it. Residual autocorrelation of candidate models without spatial error terms is evaluated through the Moran’s I statistic at distances from 100 to 1000 metres, with 100 m intervals. Spatial error terms are created based on the neighbourhood sizes with the largest Moran’s I values, that are then included in the models. This is done because we are interested in minimising bias from unmeasured spatial effects and calibrate parameters of the measured variables. Lastly, residual autocorrelation is evaluated through Moran’s I for these spatial models to ensure that biases from unmeasured spatial effects are no longer significant. All analyses are performed using R software [69] and QGIS [70].

## Results

### Descriptive analysis

**Fig 1:**
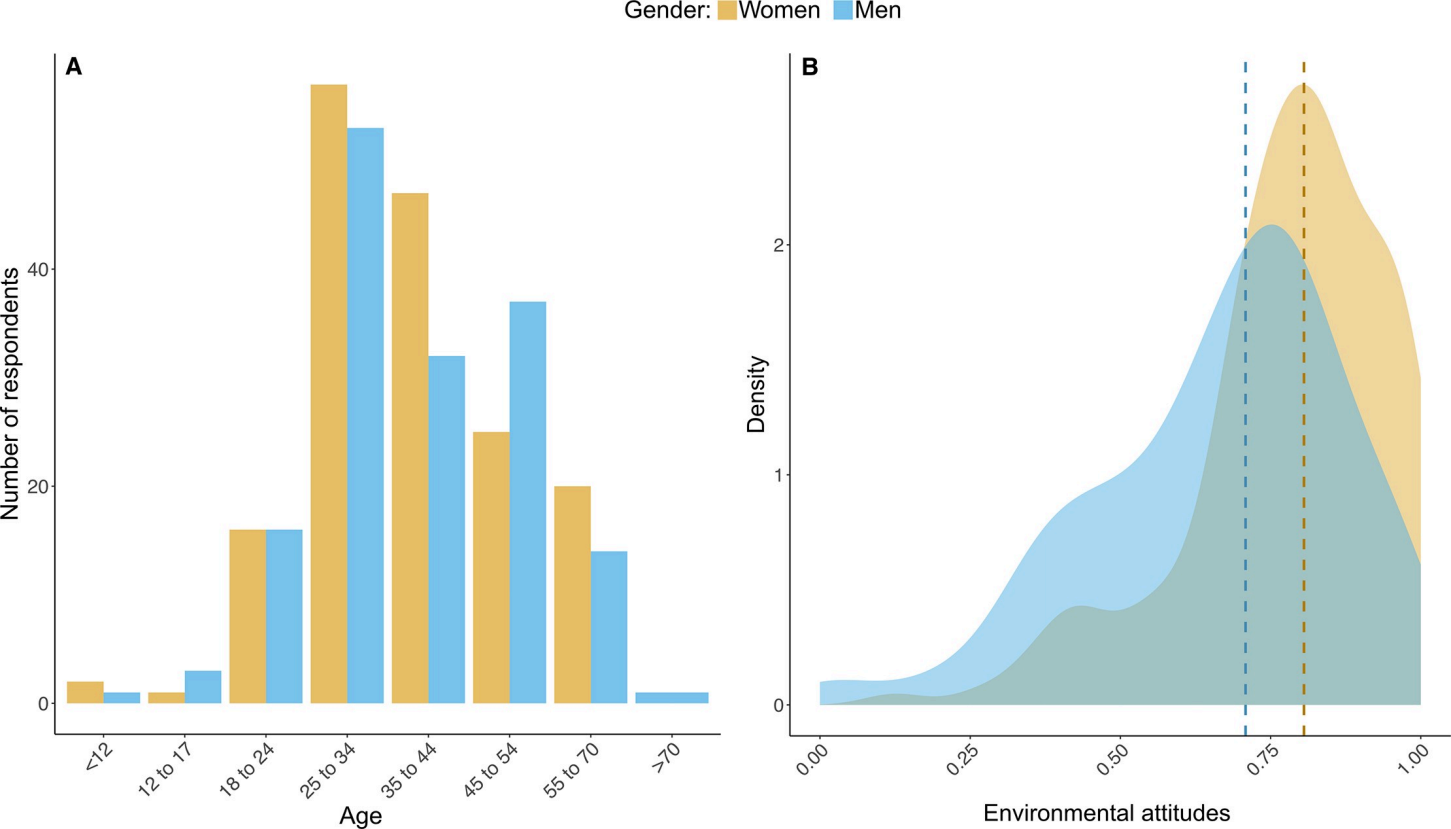
Descriptive statistics of participants. A) Distributions of age groups for each gender. B) Density distributions of EA for each gender. Dashed lines show median values for each gender.

All positive experiences that contain details about participants’ age, gender, and EA are analysed. In total, 325 respondents provide 544 positive experiences. Of these positive experiences, 100% of them have at least one indicator of restoration (see appendix 2 for details on each indicator). Participants between ages 18 and 70 make up almost the whole entirety of the sample (98%). The largest age group is 25 to 34 years old (36%), followed by 35 to 44 years old (35%) and 45 to 54 years old (20%). The distribution between women and men is fairly even (52% women) (Fig 1A). The results for EA are skewed towards higher values for both men and women (Fig 1B), but women’s EA (mean = 0.78, median = 0.81) result to be significantly higher than men’s (mean = 0.66, median = 0.71) (t(297.52) = 5.51, p<0.001). High values and gender differences are in line with existing literature and further validates our measurement of EA [71–75].

**Fig 2:**
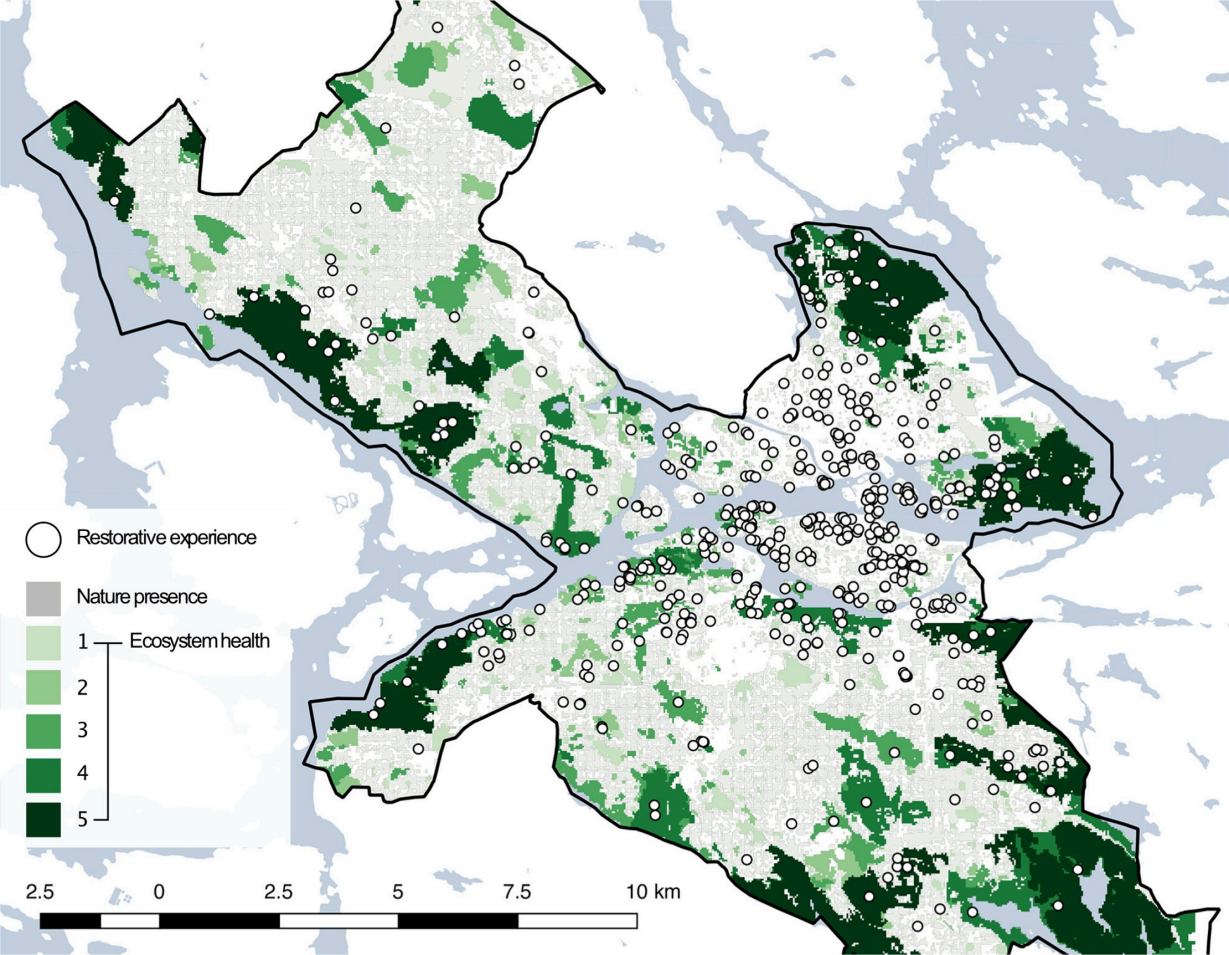
Occurrence of restorative experiences. The figure overlays the maps for nature presence, ecosystem health, and all the restorative experiences (n = 544) analysed within the boundaries of the Stockholm municipality.

Of all restorative experiences 45.7% are in natural areas and 43.0% are in areas considered having some form of healthy ecosystem (Fig 2). 16.2% are in areas with scores for EH between 0 and 1.5, 5.3% between 1.5 and 2.5, 6.6% between 2.5 and 3.5, 7.5% between 3.5 and 4.5 and 7.4% above 4.5. Hence, the first result to consider in this study is that (i) restorative experiences do not occur solely in natural environments, but can happen everywhere in the urban landscape.

### Predicting restorative experiences

Logistic regression shows how NP, NLC, EH, and EA and their statistical interactions predict restorative experiences (see Appendix 3 for details and autocorrelation analysis). There are several results worth noticing from this analysis (see Table 1 for summary). (ii) The degree of health of an ecosystem is significant to predict all restorative experiences. Independently of a person’s EA, EH predicts escaping one’s routine (OR = 3.00, 95% CI: 1.69–5.30, p<0.001), being relaxed (OR = 2.93, 95% CI: 1.48–5.79, p = 0.002), and being mindful (OR = 1.86, 95% CI: 1.03–3.36, p = 0.041). On the contrary, NP and NLC alone most often do not. When NP is modelled as an independent predictor, it does not significantly predict any restorative experiences. Among all six NLCs used, only deciduous forest results to be significant and only for feeling immersed in the place (OR = 1.75, 95% CI: 1.08–2.84, p = 0.022). These results imply that an ecologically resilient, biodiverse, and ecologically connected ecosystem is more important for restorative experiences than the mere presence of some form of natural environment. Another result to notice is that (iii) age is a significant predictor of several restorative experiences and gender of one. Older respondents show to have less restorative experiences associated with escaping one’s routine (OR = 0.07, 95% CI: 0.02–0.20, p<0.001), feeling safe (OR = 0.26, 95% CI: 0.10–0.67, p = 0.005), and being fascinated (OR = 0.13, 95% CI: 0.05–0.33, p<0.001), whereas females have more restorative experiences associated with being oneself (OR = 0.62, 95% CI: 0.43–0.90, p = 0.012).

**Table 1:** Summary of results for restorative experiences.

Restorative experience	NP^1^	NLC^2^	EH^3^	EA^4^	EA*NP	EA*EH	Age	Gender
Escaping routine	-	-	3.00***	-	-	-	0.07***	-
Being relaxed	0.30.	-	2.93**	1.11	7.67*	-	-	-
Being mindful	0.25.	-	1.86*	0.60	10.5*	-	-	-
Feeling safe	-	-	0.05*	2.08	-	62.3*	0.26**	-
Feeling immersed	-	1.75*	0.02**	0.95	-	68.0*	-	-
Being fascinated	-	-	0.01**	0.58	-	279**	0.13***	-
Being oneself	-	-	0.02*	0.89	-	193**	-	0.62*

The table shows the odds ratio values of the best-fitting models that predict each restorative experience. Colours are used to highlight the main results of this analysis. An orange background highlights the notable importance of EH over NP and NLC (ii). A green background highlights the importance of age and gender (iii). A blue background the importance of interactions between EA and either NP or EH (iv). The reference value for age is the youngest age group, and for gender it is women.

^1^ NP: Nature Presence

^2^ NLC: Natural Land Covers

^3^ EH: Ecosystem Health

^4^ EA: Environmental Attitude

p-values legend: p<0.1: .—p<0.05: *—p<0.01: **—p<0.001: ***

However, the most striking result from this analysis is that (iv) statistical interactions between EA and EH or between EA and NP predict almost all restorative experiences (except for escaping one’s routine) better than when EA, EH, and NP are independent predictors. This means that for restorative experiences the compatibility between people’s attitudes and natural environments is more relevant then the environments or the attitudes *per se*. The interaction between EA and NP is significant for experiences of being relaxed (OR = 7.66, 95% CI: 1.11–52.9, p = 0.038) and being mindful (OR = 10.5, 95% CI: 1.61–67.9, p = 0.014). Similarly, the interactions between EA and EH is significant for feeling safe (OR = 62.3, 95% CI: 1.60–2417, p = 0.027), feeling immersed in the place (OR = 68.0, 95% CI: 1.62–2860, p = 0.027), being fascinated (OR = 271, 95% CI 5.30–1380, p = 0.005), and being oneself (OR = 193, 95% CI: 4.34–8555, p = 0.007). All odds ratios reported above are robust with respect to adjusting for inclusion or exclusion of age and gender (see Appendix 3 for details). Most sensitive for adjustment is EH as a predictor for escaping one’s routine (O.R. ranging 2.41–3.00).

**Fig 3:**
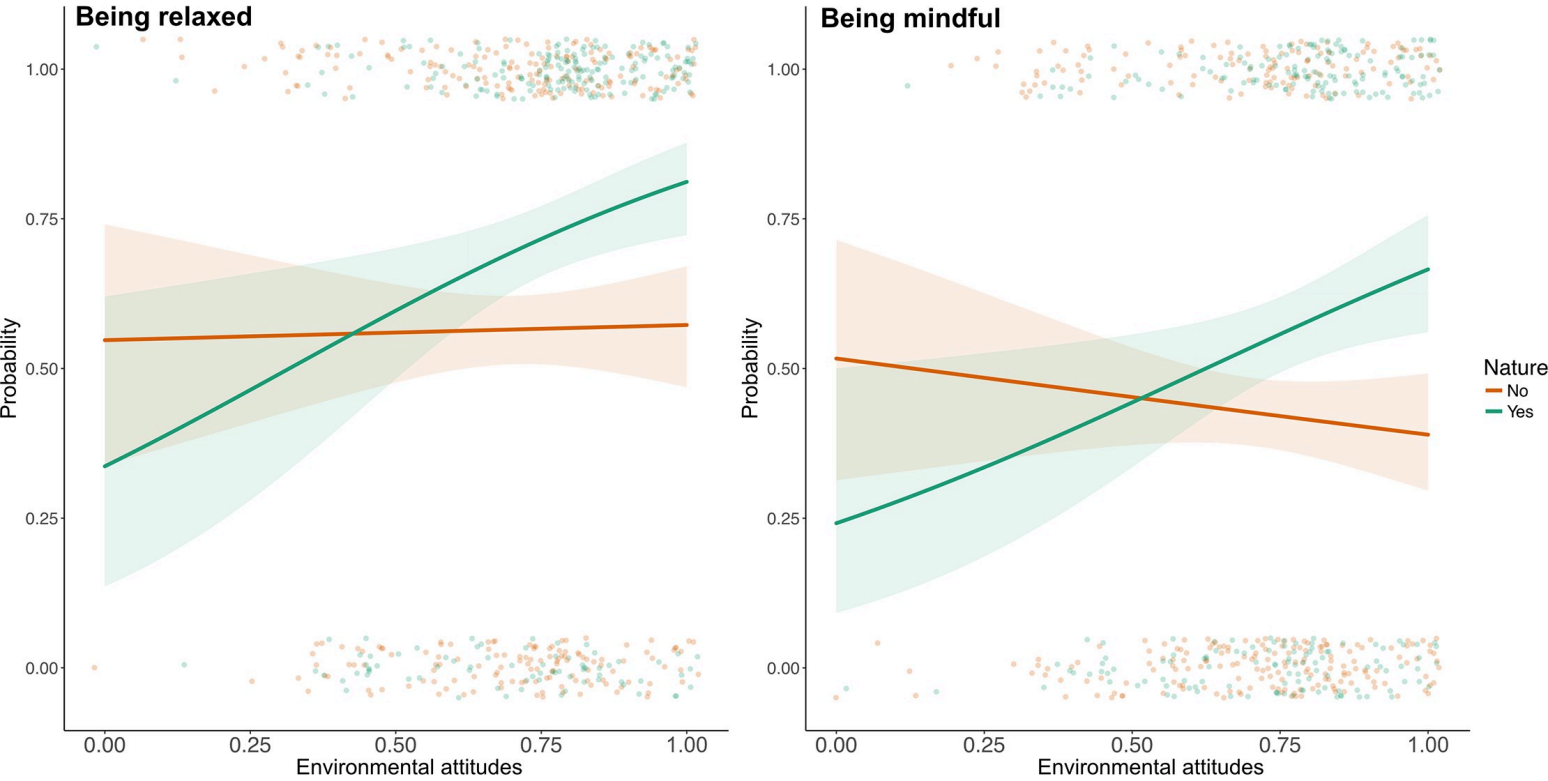
Interactions between environmental attitudes and nature presence. Probabilities that restorative experiences characterised by being relaxed and being mindful in (green lines) or outside (red lines) areas with presence of nature, in relation to respondent’s EA. Shaded areas show 95% confidence intervals of estimations. Points show actual experiences and are jittered to avoid overplotting.

The noteworthy relevance of interactions in the models requires further analysis. Hence, we transform odds ratios to probabilities and plot them to make the interactions visually understandable (Fig 3). From the graphical analysis we can observe that being relaxed and mindful are considerably more likely to happen in natural areas when people have high EA rather than low. However, this relationship ceases to be important when restorative experiences take place outside natural areas. We saw above that restorative experiences can happen everywhere, not only in natural areas. With that in mind, these results suggest that (v) when relaxation and mindfulness happen in natural environments, restorative processes are triggered by EA.

**Fig 4:**
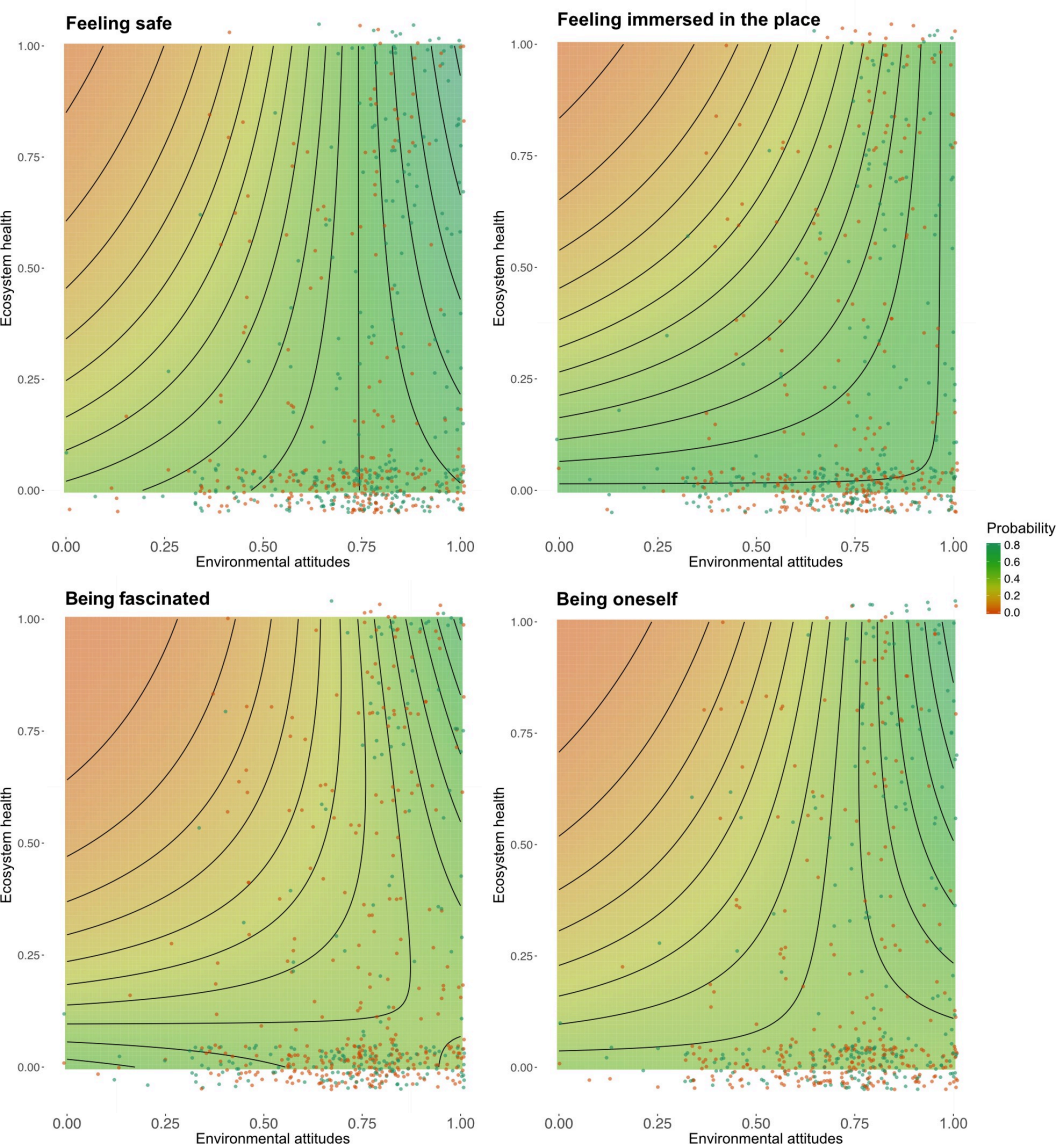
Interactions between environmental attitudes and ecosystem health. Predicted probabilities that restorative experiences are characterised by feeling safe, being immersed in the place, fascinated, or oneself, in relation to respondent’s HNC and ecosystem health at the place of the experience. Colours correspond to the probability that experiences feature the attribute and contour lines are spaced at 5 percentage points between them. Points show actual experiences and are jittered to avoid overplotting.

The analysis of interactions between EA and EH show a similar pattern of compatibility (Fig 4). Feeling safe, feeling immersed, being fascinated, and being oneself are indicators of restorative experiences that are considerably more likely to happen in natural areas when people have high EA. These results bear resemblance with what we present above for the interaction between NP and EA. When restoration from feeling safe, feeling immersed, being fascinated and being oneself happens in healthy ecosystems, restorative processes seem to be triggered by one’s attitude towards nature. We call the restorative synergy between environmental attitudes and healthy ecosystems *regenerative compatibility*.

## Discussion

### Regenerative compatibility

This paper aims to investigate how the relationship between environmental attitudes and healthy ecosystems influences restorative experiences. The results show that (i) restorative experiences can happen anywhere in the urban landscape and do not necessarily require a natural setting, but (v) when they occur in natural environments, restorative processes seem to be triggered by one’s positive attitude towards nature. (iv) The results show that including the interactions between one’s EA and natural environments makes for better predictions of almost all restorative experiences than when these variables are considered independent from each other. It is also worth noticing that, (ii) to promote restorative experiences, biodiverse and ecologically connected ecosystems are more important factors than the mere presence of natural environments. These results support the hypothesis of this study. Restorative experiences in nature happen more often when there is compatibility between people’s attitude for nature and the kind of natural environments experienced. This synergy is what we call *regenerative compatibility*. We see regenerative compatibility as a set of human-nature relationships that synergistically support healthy ecosystems, environmental attitudes, and restorative experiences.

The qualities and directionalities of interactions between healthy ecosystems, environmental attitudes, and restorative experiences might be complex. However, existing literature presents evidence of specific associations that when taken together suggest a self-reinforcing feedback loop between these variables. Reoccurring nature experiences provide a variety of health and wellbeing benefits (for reviews see [2,20,76,77]) and are known to promote EAs (for review see [78]), especially during childhood [17,79]. EAs are also known to promote nature conservation (for reviews see [80,81]), which in turn ensures the presence of nature experiences. At the same time, EAs are also known to motivate people in seeking further nature experiences [16,82–84]. Contrariwise, the lack of nature experiences in cities can promote a self-reinforcing cycle of disaffection and disengagement with the environment [85,86]. This literature suggests that the interactions between the variables representing regenerative compatibility can over time describe both regenerative and degenerative trajectories for human health and EAs, and by extension for EH.

In this study, we show that EAs moderate the restorativeness of nature experiences in the moment. We think the continuous occurrence, or not, of such experiences might be the driving factor to shape regenerative or degenerative trajectories. Hence, it might be most meaningful to consider healthy ecosystems, environmental attitudes, and restorative experiences not through their cause-effect mechanisms, but as the restorative effect being co-produced from the relation between healthy ecosystems and environmental attitudes. Regenerative compatibility is then a property that emerges from the continuous relational interaction of these variables. In summary, the relational approach underpinning regenerative compatibility offers a novel interpretation of this feedback loop by connecting trajectories of long-term change with momentary experiences.

In this study (iii) demographic factors (age and gender) are also significant predictors of several kinds of restorative experience. This result further promotes the idea that at any point in time, the restorative value of nature experiences emerges from relationships among mind, body, and environment. This relational interpretation is in line with existing literature that suggests that the psychological effect of an experience is not a direct consequence of the stimuli per se, but of the meanings that are attributed to the stimuli [28]. In relational terms, regenerative compatibility actualises restoration in nature experiences. Following the concept of embodied ecosystems [50], we argue that the regenerative or degenerative feedback loops are manifestations of the strengthening or weakening of regenerative compatibility due to its actualisation or non-actualisation in momentary experiences. It is this kind of compatible relationships between ecosystems, mind, and body that hold the potential to recreate and sustain a lost balance between human development and ecological dynamics.

As previously mentioned, our participants are not a representative sample of the Swedish population. For example, the descriptive analysis showed that 71% of respondents are from 25 to 44 years old. We also focus specifically on city dwellers and nature in urban environments. Hence, we are wary of concluding that our results are universally generalisable or applicable beyond the urban context. However, our results are supported by theory, so verifying the regenerative compatibility with different samples in different settings and through longitudinal designs is a promising and important task for future research. Within the context of Western urbanised societies, we believe that regenerative compatibility has the potential for being used as a leverage point for a sustainable and healthy urban living [87].

### Regenerative compatibility, ecosystem health, and human health

Regenerative compatibility might be a key aspect to promote healthy and sustainable urban living in the future because it could resolve several issues where ecosystem and human health seem at odds. For example, Gatersleben and Andrews [88] show that perceived safety in natural environments is important for their restorative function. But while they study perceived safety as a consequence of the environment’s physical structure (the visibility it affords), we study it as emergent from the interaction between a person’s mind and the physical environment. Our results show that including this interaction improves predictions as compared to when the environment is modelled as an independent predictor. Being comfortable in nature is to a large extent a learnt ability [17,89]. This ability might be crucial to safeguard from a public health perspective, as the mere presence of residential green space in childhood is strongly associated with lower occurrences of psychiatric disorders in new generations [31].

Van Heezik and Brymer [90] note that trade-offs related to physiological aspects of health might exist, for example when higher levels of biodiversity cause problems related to pollen. However, exposure to biodiverse nature among adolescents is linked to reduced allergic dispositions [91]. In the public health literature, reduced contact with environmental features, biodiversity, and microbiota, is well-known to lead to immunodeficiencies [91,92]. This begs the question whether this issue could be more sustainably addressed in the long term by promoting human-nature interactions rather than by limiting them, especially when simultaneously considering other physical health benefits of nature presence in cities. For example, local green areas provide air purification services of remarkable value to the health of urban populations [93].

We agree with many others [7,94] that dissolving the fictitious dichotomy between people and nature is the only viable solution for having healthy people on a healthy planet. Ultimately, indications that what is best for human health is at odds with what is best for ecosystems might be symptomatic of a deeper need to reorient sustainability science towards, first and foremost, promoting sustainable relationships between people and planet [44,87].

### Policy recommendations

Regenerative compatibility suggests that human habitats that are ecologically sustainable and support healthy and sustainable living do not need to be utopian—rather the opposite. This study contributes to the vast literature that directly links public health and wellbeing benefits to the availability of nature experiences in cities [2,20]. However, our results specifically suggest that the positive effects of nature interactions are enabled by positive environmental attitudes (v) and amplified by the ecological health of the ecosystem (ii). Consequently, future policies for sustainable urban development should consider environmental education, city design, and urban ecology jointly. Given the non-linear behaviour of self-reinforcing dynamics, multifaceted solutions that unify these areas can have great leverage for rapid changes with long-term impact.

Multifaceted policy interventions might provide synergistic and long-lasting benefits that counter the limitations of the more common reductionist and short-term approaches. For example, Stanley et al. [95] argue that policies that promote public health through the urban green infrastructure pose a threat to urban ecosystems, as it requires urban nature to become more ‘people-friendly’. Examples of this are the construction of walkways, clearing of understory vegetation and preference for flat open spaces. Yet, we found that people with high environmental attitudes do not seek out these environments for restoration (ii). Wild areas can be as relaxing as manicured environments when people are more connected with nature [96]. Learning to be comfortable and appreciating different natural environments is a function of reoccurring nature experiences [17,89]. Given that a sustainable human habitat has to exist within ecologically sustainable and resilient ecosystems, a long-term solution might have to address what is culturally assumed to be ‘people-friendly’ nature, rather than isolating issues of ecosystem conservation and urban nature experiences from each other. The value of urban nature for public health is amplified when combined with environmental education. Supporting inhabitants’ wellbeing, conserving local flora and fauna, and promoting environmental education should be seen as different requirements of the same design intervention.

Integrating experiences of different kinds of nature, from the wild to the manicured, with urban life requires nature to cover large geographical areas in cities. As nature and buildings compete for space in cities, policies that promote regenerative compatibility might be at odds with compact city development. In order to respond to global environmental challenges, increasing urban densification is a recognised spatial solution to reduce greenhouse gas emissions from transportation and increase energy efficiency [97–99]. However, when social dynamics are taken into consideration, urban densification is not a driving variable to reduce carbon footprint [100]. For example, electricity consumption per capita is not related to compact urban form per se [101]. Increased urban density is linked to increased weekend trips and short- and long-haul air travel [102]. Once more, the potential to respond to the climate crisis by maximising one single attribute, such as urban densification, is limited [103] and policy interventions have to be multifaceted.

Realising living environments for humans that combine energy efficiency with healthy and expansive ecosystems requires a shift in focus from a simplistic ‘dense vs. green’ framing [104]. Policy interventions have to be developed in conjunction with human experiences and social values [105]. The importance of nurturing shared values for nature for a sustainable future is remarked by many academic authors [7,44,106] and a few noted its particular relevance in the context of developing sustainable human habitats [94,107,108]. In cities, policies have to simultaneously address climate change, disrupted ecosystem services, unhealthy habits, and unsustainable lifestyles. These objectives cannot be considered in separation from each other. Ultimately, the human habitat has to exist within ecologically sustainable and resilient ecosystems. Separating human and natural living environments might be an obsolete custom in city design that have no place in shaping future sustainable human habitats.

Policies that exploit synergies like regenerative compatibility and promote dynamic and holistic interventions, rather than static and isolated ones, might be better suited to couple healthy living with an urban development supportive of the biosphere. Regenerative compatibility might be just one of several potential regenerative dynamics valuable to design sustainable human habitats. Nevertheless, it suggests a way towards sustainable human development through the regeneration of human-nature relationships rather than through the implementation of atomistic solutions.

## Conclusion

The severe global environmental challenges that cities face demand human habitats that support both healthy people and a sustainable biosphere. In this study, we find a synergy between healthy ecosystems, environmental attitudes, and restorative experiences that we call *regenerative compatibility*. Restorative experiences in nature are more likely to happen in healthy ecosystems and among people with positive environmental attitudes. This could prove to be a general synergy that is worth further academic exploration and practical application in nature-based solutions. We believe that sustainable human habitats are best understood as relational systems that intertwine psychological, social, and environmental variables. Nurturing regenerative compatibility could help to dissolve fictitious dichotomies that still exist between people and nature, between healthy humans and a healthy biosphere, and ultimately between natural habitats and human habitats. Thus, interventions to promote future sustainable cities ought to address people’s health, environmental education, and urban ecology simultaneously. Such approaches are central to the shift from a static and compartmentalised view of sustainability to one that is holistic, dynamic, and regenerative.

## Supporting information

S1 Table Table of items for environmental attitudes. Table with items used in the survey “Var är ditt Stockholm?” to assess environmental attitudes.
(DOCX)

S2 Table Description of restorative experiences. Table with number of each typology of restorative experiences and percentage on the total amount of experiences analysed.
(DOCX)

S3 Table Regressions and spatial autocorrelation results. Table of results from the regression and spatial autocorrelation analysis for each restorative experience.
(DOCX)
