# Supplementary material for: The regenerative compatibility: A synergy between healthy ecosystems, environmental attitudes, and restorative experiences
Source: PLoS One. 2020 Jan 7;15(1):e0227311. doi: 10.1371/journal.pone.0227311 (PMC6946585; doi:10.1371/journal.pone.0227311)
Supplement: S1 Table — Table with items used in the survey “Var är ditt Stockholm?” to assess environmental attitudes. (DOCX) [file pone.0227311.s001.docx]

**S1 Table. Items used to assess environmental attitudes.** Table of items used in the survey “Var är ditt Stockholm?” to assess environmental attitudes. For each indicator, the related sentence utilised in the survey is reported, and the original scale from which the sentence is taken.

| Indicators of  Environmental Attitudes | Item in “Var är ditt Stockholm?” | Original scale of reference |
| --- | --- | --- |
| Enjoyment of nature | I like go outside and enjoy nature, even in unpleasant weather | Nature Relatedness Scale ^1^ |
| Empathy for animals | I feel empathy for the feelings of animals | Connection to Nature Index ^2^ |
| Domination over nature | Humans were meant to rule over the rest of nature | New Environmental Paradigm ^3^ |
| Identification with nature | I think of myself as part of all living things and the earth. I am not different from nature. | Environmental Identity ^4^ |
| Identification with nature | Being a part of nature is an important part of who I am | Environmental Identity |
| Environmental awareness | Human well-being depends on plants and animals | Connection to Nature Index |
| Sense of responsibility | Taking care of nature is important to me | Love and Care for Nature ^5^ |
| Environmental concern | I am concerned about the state of the environment | Environmental Concern ^6^ |

Bibliography of the original scales of references for each item:

1. Nisbet, E. K., Zelenski, J. M. & Murphy, S. a. The Nature Relatedness Scale: Linking Individuals’ Connection With Nature to Environmental Concern and Behavior. Environ. Behav. 41, 715–740 (2008).

2. Cheng, J. C.-H. & Monroe, M. C. Connection to nature: Children’s affective attitude toward nature. Environ. Behav. 44, 31–49 (2012).

3. Dunlap, R. E. & Van Liere, K. The new environmental paradigm. J. Environ. Educ. 9, 10–19 (1978).

4. Clayton, S. Environmental Identity: A Conceptual and Operational Definition. in Identity and the Natural Environment: The Psychological Significance of Nature (eds. Clayton, S. & Opotow, S.) 45–66 (MIT Press, 2003).

5. Perkins, H. E. Measuring love and care for nature. J. Environ. Psychol. 30, 455–463 (2010).

6. Ellis, R. J. & Thompson, F. Culture and the environment in the Pacific Northwest. Am. Polit. Sci. Rev. 91, 885–897 (1997).
