# Supplementary material for: The regenerative compatibility: A synergy between healthy ecosystems, environmental attitudes, and restorative experiences
Source: PLoS One. 2020 Jan 7;15(1):e0227311. doi: 10.1371/journal.pone.0227311 (PMC6946585; doi:10.1371/journal.pone.0227311)
Supplement: S2 Table — Table with number of each typology of restorative experiences and percentage on the total amount of experiences analysed. (DOCX) [file pone.0227311.s002.docx]

**S2 Table. Description of restorative experiences.** Table with number of each typology of restorative experiences and percentage on the total amount of experiences analysed

| Restorative indicator | number | Percentage on total |
| --- | --- | --- |
| being mindful | 265 | 48.71% |
| feeling immersed | 253 | 46.51% |
| escaping routine | 149 | 27.39% |
| being oneself | 240 | 44.11% |
| feeling safe | 288 | 52.94% |
| being fascinated | 208 | 38.24% |
| feeling relaxed | 341 | 62.68% |
