# Supplementary material for: The regenerative compatibility: A synergy between healthy ecosystems, environmental attitudes, and restorative experiences
Source: PLoS One. 2020 Jan 7;15(1):e0227311. doi: 10.1371/journal.pone.0227311 (PMC6946585; doi:10.1371/journal.pone.0227311)
Supplement: S3 Table — Table of results from the regression and spatial autocorrelation analysis for each restorative experience. (DOCX) [file pone.0227311.s003.docx]

**S3 Table. Regressions and spatial autocorrelation results.** Table of best fitting models from the regression and spatial autocorrelation analysis for each restorative experience. O.R. range describes the range that odds ratios for variables take in models adjusting or not adjusting for age and gender, to indicate the robustness of effect sizes. Neighbourhood describes the size of the neighbourhood for calculating the autocorrelation matrix included in the spatial model, Moran’s I describes the autocorrelation of residuals from the spatial model (non-significant means that residual autocorrelation has been dealt with), and ΔAIC means the relative improvement in model fit between non-spatial and spatial models.

|  | Being relaxed | |  |  | Being mindful | |  |  |
| --- | --- | --- | --- | --- | --- | --- | --- | --- |
|  | O.R. | 95% C.I. | p-value | O.R. range | O.R. | 95% C.I. | p-value | O.R. range |
| Nature Presence | 0.30 | 0.07, 1.28 | 0.104. | 0.29-0.30 | 0.25 | 0.06, 1.03 | 0.055. | 0.24-0.25 |
| Ecosystem Health | 2.93 | 1.48, 5.79 | 0.002** | 2.91-2.95 | 1.86 | 1.03, 3.36 | 0.041* | 1.86-1.91 |
| SA | 1.11 | 0.34, 3.58 | 0.864 | 0.99-1.11 | 0.60 | 0.19, 1.91 | 0.385 | 0.59-0.67 |
| **Interactions** |  |  |  |  |  |  |  |  |
| NP*SA | 7.67 | 1.11, 52.94 | 0.039* | 7.67-8.13 | 10.47 | 1.61, 67.92 | 0.014* | 10.32-11.40 |
| Autocorrelation  matrix |  |  | 0.013* |  |  |  | 0.067 |  |
| Neighbourhood | 700 m |  |  |  | 500 m |  |  |  |
| Moran’s I | 0.011 |  | 0.291 |  | 0.045 |  | 0.064 |  |
| ΔAIC | -4.38 |  |  |  | -1.39 |  |  |  |

|  | Feeling fascinated | |  |  | Being oneself | |  |  |
| --- | --- | --- | --- | --- | --- | --- | --- | --- |
|  | O.R. | 95% C.I. | p-value | O.R. range | O.R. | 95% C.I. | p-value | O.R. range |
| Ecosystem health | 0.01 | 0.00, 0.30 | 0.007** | 0.01 | 0.02 | 0.00, 0.45 | 0.012* | 0.02 |
| SA | 0.58 | 0.20, 1.68 | 0.317 | 0.57-0.61 | 0.89 | 0.30, 2.66 | 0.840 | 0.89-1.39 |
| Age | 0.13 | 0.05, 0.33 | <0.001*** | 0.13 |  |  |  |  |
| Gender (male) |  |  |  |  | 0.62 | 0.43, 0.90 | 0.012* | 0.62 |
| **Interactions** |  |  |  |  |  |  |  |  |
| EH*SA | 279 | 5.53, 1413 | 0.005** | 271-279 | 193 | 4.38, 8555 | 0.007** | 165-193 |
| Autocorrelation matrix |  |  | 0.010** |  |  |  | 0.006** |  |
| Neighbourhood | 900 m |  |  |  | 400 m |  |  |  |
| Moran’s I | 0.015 |  | 0.203 |  | 0.012 |  | 0.347 |  |
| ΔAIC | -4.73 |  |  |  | -5.85 |  |  |  |

|  | Feeling immersed | |  |  | Feeling safe | |  |  |
| --- | --- | --- | --- | --- | --- | --- | --- | --- |
|  | O.R. | 95% C.I. | p-value | O.R. range | O.R. | 95% C.I. | p-value | O.R. range |
| Ecosystem health | 0.02 | 0.00, 0.37 | 0.011* | 0.02 | 0.05 | 0.00, 0.94 | 0.042* | 0.05 |
| Deciduous forest | 2.27 | 1.12, 4.60 | 0.022* | 2.27-2.28 |  |  |  |  |
| SA | 0.95 | 0.34, 2.64 | 0.917 | 0.95-0.96 | 2.08 | 0.74, 5.86 | 0.168 | 1.97-2.41 |
| Age |  |  |  |  | 0.26 | 0.10, 0.67 | 0.005** | 0.26 |
| **Interactions** |  |  |  |  |  |  |  |  |
| EH*SA | 68.1 | 1.62, 2861 | 0.027* | 67.8-68.1 | 62.3 | 1.60, 2417 | 0.027* | 56.4-62.3 |
| Autocorrelation matrix |  |  | 0.469 |  |  |  | 0.007** |  |
| Neighbourhood | 900 m |  |  |  | 400 m |  |  |  |
| Moran’s I | 0.024 |  | 0.108 |  | 0.029 |  | 0.192 |  |
| ΔAIC | 1.47 |  |  |  | -5.60 |  |  |  |

|  | Escaping routine | |  |  |
| --- | --- | --- | --- | --- |
|  | O.R. | 95% C.I. | p-value | O.R. range |
| Ecosystem health | 3.00 | 1.69, 5.30 | <0.001*** | 2.41-3.00 |
| Deciduous forest |  |  |  |  |
| SA |  |  |  |  |
| Age | 0.07 | 0.02, 0.20 | <0.001*** | 0.07 |
| Autocorrelation matrix |  |  | 0.002** |  |
| Neighbourhood | 900 m |  |  |  |
| Moran’s I | -0.008 |  | 0.581 |  |
| ΔAIC | -7.73 |  |  |  |
